# High-throughput Measurement of *Dictyostelium discoideum* Macropinocytosis by Flow Cytometry

**DOI:** 10.3791/58434

**Published:** 2018-09-10

**Authors:** Thomas Williams, Robert R. Kay

**Affiliations:** ^1^MRC-Laboratory of Molecular Biology, Cambridge, UK

**Keywords:** Biology, Issue 139, *Dictyostelium discoideum*, flow cytometry, macropinocytosis, high-throughput, endocytosis, single-cell resolution, fluid uptake

## Abstract

Large-scale non-specific fluid uptake by macropinocytosis is important for the proliferation of certain cancer cells, antigen sampling, host cell invasion and the spread of neurodegenerative diseases. The commonly used laboratory strains of the amoeba *Dictyostelium discoideum *have extremely high fluid uptake rates when grown in nutrient medium, over 90% of which is due to macropinocytosis. In addition, many of the known core components of mammalian macropinocytosis are also present, making it an excellent model system for studying macropinocytosis. Here, the standard technique to measure internalized fluid using fluorescent dextran as a label is adapted to a 96-well plate format, with the samples analyzed by flow cytometry using a high-throughput sampling (HTS) attachment.

Cells are fed non-quenchable fluorescent dextran for a pre-determined length of time, washed by immersion in ice-cold buffer and detached using 5 mM sodium azide, which also stops exocytosis. Cells in each well are then analyzed by flow cytometry. The method can also be adapted to measure membrane uptake and phagocytosis of fluorescent beads or bacteria.

This method was designed to allow measurement of fluid uptake by *Dictyostelium* in a high-throughput, labor and resource efficient manner. It allows simultaneous comparison of multiple strains (*e.g.* knockout mutants of a gene) and conditions (*e.g.* cells in different media or treated with different concentrations of inhibitor) in parallel and simplifies time-courses.

**Figure:**
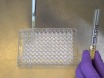


## Introduction

Large-scale non-specific fluid uptake by macropinocytosis is important in several biological contexts^1^, including antigen sampling by immune cells^2^, pathogen entry into host cells^3^, cancer cell proliferation^4^ and the spreading of prion diseases^5^. In mammalian and *Dictyostelium *cells, actin^6^,^7^, PI(3,4,5)P3^8^,^9^,^10^ (although the exact nature of the lipid differs between the two^11^), activated Ras^12^,^13^, and activated Rac^14^,^15^ are important for efficient fluid uptake by macropinocytosis, although there remain many unanswered questions about how the macropinocytic patch is formed, organized and eventually internalized. Discovering more proteins important for macropinocytosis, and subsequent determination of how they are important in the various biological contexts, will give a more comprehensive understanding of macropinocytosis and potentially allow development of targeted treatments for a range of conditions.

*Dictyostelium *is an ideal model system for studying macropinocytosis. The high level of constitutive macropinocytosis in standard laboratory strains means that fluid uptake is over 90% due to macropinocytosis^6^. This allows macropinocytosis to be measured solely by determining fluid uptake, unlike mammalian cells where the proportion of fluid uptake due to macropinocytosis is much lower. That macropinocytosis is so well defined and easily visualized^12^ in this system similarly offers distinct advantages for investigating core conserved components of the macropinosome over other systems where there may be multiple regulatory signals^16^,^17^.

The standard technique used to measure macropinocytosis by mammalian cells involves fixing cells after pulsing with dextran for a short period of time followed by microscopy to determine the area of a cell that is occupied by dextran-positive vesicles^18^. This technique does not however account for the possibility of macropinosomes shrinking upon entering the cell, which has been reported in *Dictyostelium*^19^, and only takes into account single planes of the cell, meaning the volume internalized is unclear. An alternative technique, of counting the number of macropinosomes internalized in a given time, has the same downsides^20^. Using *Dictyostelium* avoids these issues; however, existing techniques for measuring fluid uptake by *Dictyostelium* are relatively labor intensive, using a large amount of both cells and dextran^21^. Cells are shaken at high density in fluorescent dextran and samples removed at various time-points for determination of the internalized fluorescence using a fluorimeter. Cells prepared this way can be analyzed by flow cytometry to gain single cell, rather than population-level, resolution^22^, although this remains low-throughput.

Here, the standard technique to measure internalized fluid using fluorescent dextran as a label is adapted to a 96-well plate format, with the samples analyzed by flow cytometry using a high-throughput sampling (HTS) attachment. Cells are fed non-quenchable fluorescent dextran for a pre-determined length of time, washed by immersion in ice-cold buffer and detached using 5 mM sodium azide, which also stops exocytosis. Cells in each well are then analyzed by flow cytometry. This method was designed to overcome the limitations of the above methods and allow simultaneous comparison of the fluid uptake of large numbers of strains/conditions while using fewer resources and reducing the labor involved.

## Protocol

### 1. Preparation of Cells and Materials

1. Cultivate cells either on SM agar plates in conjunction with bacteria such as *Klebsiella aerogenes*, or in nutrient medium such as HL5 as described^23^. NOTE: Growing knockout mutants that are defective in macropinocytosis on bacteria helps prevent accumulation of secondary mutations that may increase the rate of macropinocytosis. Keep the passage number below 4 after plating cells from stocks for optimum results. Mutants should be stored as frozen stocks as soon as possible after isolation.
2. To cultivate cells on SM agar plates, grow *K. aerogenes* to confluence in SM medium. Add 200–300 μL of bacteria onto an SM agar plate and spread. Take a sterile loop of cells (when transferring from another bacterial plate) and spread at one edge of the plate. Incubate at 22 °C for up to a week.
3. Dissolve Tetra-methyl-rhodamine isothiocyanate (TRITC-) dextran to 50 mg/mL in water, filter using 0.22 μm filters into 1 mL aliquots and store at -20 °C. Aliquots can be kept indefinitely. NOTE: 155 kDa is the typical size used with this method, as it can be bought cheaply in bulk, but smaller dextrans measure macropinocytosis just as effectively in *Dictyostelium*^24^. Different non-quenchable dextrans, such as cascade blue or Alexa-647, are alternatives if different fluorophores are required.

### 2. Converting Qualitative Measurements into Quantitative (Optional)

1. **Perform a fluid uptake assay using cells in suspension.**
  1. Pellet axenically growing cells at 300 x g for 3 min, discard the supernatant and resuspend in nutrient medium to 1x10^7^ cells/mL. Add TRITC-dextran to 0.5 mg/mL and shake at 180 rpm, 22 °C.
  2. Dilute 0.8 mL samples into 0.7 mL of ice-cold KK_2_ at 0, 30, 60, 90, 120 min. Wash once in 1.5 mL of ice-cold KK_2 _buffer^23^ in a benchtop centrifuge, resuspend to 1 mL in the same buffer and store on ice. NOTE:****washing immediately or once all samples have been collected yields equivalent results.
2. **Determine the volume of fluid internalized by the cells.**
  1. Set the fluorimeter to measure fluorescence using an excitation wavelength of 544 nm and emission wavelength of 574 nm with a 10 nm slit. Set up a dilution series of TRITC-dextran and use it to measure the fluorescence for given volumes of dextran. Create a calibration curve.
  2. Measure the fluorescence internalized by the cells in section 2.1 using 0.9 mL of the cell suspension. Calculate the volume of fluid internalized per cell using the calibration curve^24^.
3. Dilute the remainder of the cells from each time point separately into 0.5 mL ice cold KK_2_. Filter through a 70 µm cell strainer into 5 mL polystyrene flow cytometry tubes. Set the flow cytometer so that cells from each time point are distinct from each other and record their fluorescence (see sections 3.4 and 3.5).
4. Measure the fluorescence of calibration beads in the flow cytometer using the settings above and record it. NOTE: This is the reference for all future fluorescence measurements on the flow cytometer: before using again run the beads and adjust the settings so they have the same fluorescence. This will give a very narrow defined peak. Putting a gate around the fluorescence peak can make this easier.
5. Repeat three times and plot the cell fluorescence data obtained in sections 2.2 and 2.3 against each other. Plot a line of best fit and use the equation to convert the qualitative data from the flow cytometry into quantitative units.

### 3. Measuring Fluid Uptake

**Figure:**
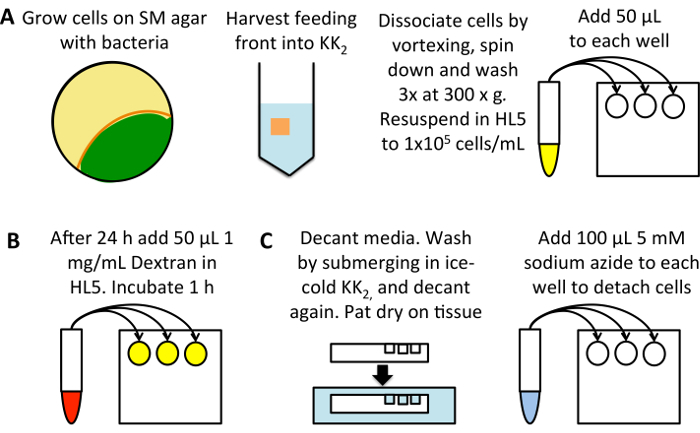

**Figure 1: Schematic of high-throughput measurement of macropinocytosis. (A)** Grow *Dictyostelium *on an SM plate seeded with *K. aerogenes* bacteria (yellow). Harvest cells from the feeding front (orange), avoiding cells that are already developed (green), into 25 mL of KK_2 _buffer. Vortex to dissociate, pellet by 3 min centrifugation at 300 x *g*, then wash 3 times in 50 mL of KK_2 _buffer, discarding the supernatant each time. Resuspend to 1 x 10^5^ cells/mL in HL5 growth medium and add 50 µL into three wells per sample of a flat bottom 96-well plate. Incubate at 22 °C for 24 h. **(B)** Dilute TRITC-dextran to 1 mg/mL in HL5 growth medium from a stock solution of 50 mg/mL. Add 50 µL to each sample (excluding the 0 min uptake control wells), and incubate at 22 °C for 1 h, after which add the dextran to the 0 min uptake control. **(C) **Immediately decant the media, pat the plate on a tissue to remove excess medium and submerge in a bath of ice-cold KK_2 _buffer, filling the wells to wash. Decant the buffer and pat dry again. Add 100 µL of 5 mM sodium azide dissolved in KK_2_MC to detach the cells. Take to flow cytometer for measurement of internalized fluorescence. Please click here to view a larger version of this figure.

1. **Set up a flat-bottomed 96-well tissue-culture plate (Figure 1A). **Use cells grown on bacteria and to transfer them into HL5 medium (containing 100 µg/mL of dihydrostreptomycin, 100 µg/mL ampicillin and 50 µg/mL kanamycin) 24 h before the assay; this allows macropinocytosis to be upregulated from the low levels seen in cells grown on bacteria^24^. Alternatively, dilute cells directly from axenic culture, incubating for 24 h before the assay, to reduce errors due to the cells being diluted from different densities.
  1. Harvest cells from the feeding front into 25 mL of KK_2_ in a 50 mL centrifuge tube. Dissociate cells by vortexing and pellet at 300 x *g* for 3 min*. *Discard the supernatant, resuspend the pellet and wash 3 times at 300 x *g* for 3 min in 50 mL of KK_2_, discarding the supernatant each time to remove the bacteria.
  2. Determine the cell density (using a hemacytometer or other cell counting system) and dilute into HL5 containing antibiotics to 1 x 10^5^ cells/mL. Pipette 50 μL into each well, using three wells for each condition. Incubate at 22 °C for 24 h. Remember to set up a 0 min uptake control. NOTE: An alternative medium, *e.g.* SIH, VL6 can be used instead.
2. **Load cells with TRITC-dextran (Figure 1B).**
  1. Dilute the dextran to 1 mg/mL in the medium used (this can be increased to 2.5–5 mg/mL when assessing cells with very low uptake). Add 50 μL to each well (giving a final concentration of 0.5 mg/mL TRITC-dextran) and return the plate to 22 °C for 1 h, as this allows significant dextran accumulation but exocytosis of dextran has not yet begun. NOTE:****A repeater pipette allows this step to be done more rapidly, reducing error.
3. **Prepare cells for flow cytometry (Figure 1C).**
  1. Immediately prior to washing, add 50 μL dextran-containing media to the 0 min uptake controls.
  2. Decant the medium and pat dry on a tissue. Wash by submerging the plate in ice-cold KK_2_, then decant. NOTE:****some strains with adherence defects may become detached during this step, *e.g.* a knockout of both homologs of Talin (*talA-/talB-*)^25^. Use caution when working with cell lines that attach poorly and aspirate media if required.
  3. Add 100 μL of ice-cold 5 mM sodium azide dissolved in KK_2_MC (KK_2_ + 2 mM MgSO_4_ and 100 μM CaCl_2_). CAUTION: Sodium azide is extremely toxic. Use a dust mask and safety glasses when working with the powder. Always wear gloves and lab coat. Avoid heat and do not mix with acid. NOTE: Cells rapidly detach, and exocytosis is prevented^24^. A microscope can be used to check this.
4. **Measure fluid uptake by flow cytometry**
  1. If planning to convert relative values to absolute ones, use beads to standardize flow cytometer settings (see section 2). Alternatively, use cells loaded with dextran as in section 2 to ensure that the forward scatter and side scatter are set appropriately to isolate cells (Figure 2A), ensuring the machine is not blocked, (Figure 2B) and adjust the parameters to measure the internalized fluorescence (Figure 2C).
  2. Attach high-throughput sampling system and add plate. Set up a protocol to measure the fluorescence (for TRITC-dextran use a yellow-green laser to excite and measure in the 582 nm channel or similar) of up to 65 μL of the cell suspension and run.
5. **Analyze flow cytometry results**
  1. Gate on the cells using forward scatter and side scatter (Figure 2A). Calculate the median fluorescence of the cells using the statistics option. Set these parameters using one sample and apply to all samples. NOTE: The forward scatter/side scatter profiles can vary between samples when using different mutants or inhibitors.
  2. Determine the mean of the three wells for each condition and subtract the 0 min uptake control. Either convert to quantitative values using the equation determined in section 2 or normalize to the control.

### 4. Performing Fluid Uptake Time-courses

1. Set up cells as in section 3, with three wells for each time point and strain/condition.
2. Add dextran labelled medium to the different wells sequentially. A typical time-course measures fluid uptake at 0, 30, 60, 90, 120 and 180 min. Add dextran-containing medium for the 180 min time point wells, followed 60 min later by adding it to the wells for the 120 min time point, *etc.* (Figure 3A).
3. At 0 min, add 50 μL of dextran-containing medium and immediately decant and wash the plate as in section 3.3. Analyze as described in section 3.5.

### 5. Dose Response Curves

1. Set up cells as described in section 3, with three wells for each strain and condition.
2. Dilute the compound of interest into medium containing 1 mg/mL dextran at *double the desired maximum final concentration *of the compound. Prepare a second tube containing medium with dextran with the same proportion of vehicle as the first. Vortex to mix.
3. Create a dilution series of the compound of interest in 200 μL dextran containing medium per sample (Figure 4A). Vortex to mix. Add 50 μL of the medium to each sample well for 1 h.
4. Wash and analyze as described in section 3.3 onwards.

### 6. Phagocytosis and Membrane Uptake

1. To measure membrane uptake, prepare the cells as described in section 3. Add 50 μL of medium containing 20 μM FM 1-43 for a final concentration of 10 μM. After loading, wash and prepare cells for flow cytometry as in section 3.3. Measure in the 585 nm channel or similar, using a blue laser to excite. NOTE: As membrane trafficking is more rapid than fluid-phase, 10 min is a more appropriate time point to use than 1 h^26^. Alternative dyes that fluoresce only when incorporated into the membrane can be used instead.
2. **Use either fluorescently labelled beads or bacteria to measure phagocytosis. **Fluorescent yeast cannot be used, as they are inseparable from *Dictyostelium* by forward and side scatter^24^. Set up cells as described in section 3 and analyze as described^27^.
  1. To measure phagocytosis of beads add yellow-green labelled beads as described in section 3.2 to a final concentration of 5 x 10^7^ beads/mL (1.75 and 2 μm) or 1 x 10^8^ beads/mL (1 and 1.5 μm) for 1 h before washing and preparing for flow cytometry as in section 3.3. Measure in the 525 nm channel or similar, using a blue laser to excite. Note: Higher concentrations will lead to cell detachment.
  2. To measure phagocytosis of bacteria, add Texas-Red labelled *Escherichia coli* bioparticles as described in section 3.2 to 1 x 10^8^ bacteria/mL for 1 h before washing and preparing for flow cytometry as in section 3.3. Measure in the 610 nm channel or similar, using a yellow-green laser to excite.

## Representative Results

Once the technique has been performed and cells are loaded with dextran and ready for analysis (Figure 1), ensure the flow cytometer is not blocked and adjust the forward scatter/side scatter profile to look like the cells shown in Figure 2A. If the machine is blocked, it will look more like that shown in Figure 2B and must be unblocked before continuing. Ensure the parameters show control axenic cells have high internalized dextran fluorescence at longer time points and low internalized fluorescence at shorter ones (Figure 2C).

When looking for differences between mutants, it is likely that there will be one of three phenotypes. The mutants could have normal fluid uptake, they could have a partial defect, or fluid uptake could be completely abolished. Figure 2D shows a strain with normal fluid uptake, in this case the standard laboratory strain Ax2, a mutant with a ~50% decrease in fluid uptake (Ax2 *rasG-*14) and one with abolished fluid uptake (Ax3 *gefB-*28). Obtain the average median fluid uptake (section 3.5) and use it to either calculate the volume of fluid internalized (as in Figure 3B) or compare the data to a control (as in Figure 4B****and** 4C**).

When performing a fluid uptake time course, as in Figure 3A, the internalized fluorescence should increase for 60–90 min, after which the dextran begins to be exocytosed and a plateau is reached (Figure 3B). Using 60 min as the standard time point when comparing macropinocytosis in different mutants/conditions therefore allows a good signal to be achieved, and no signal is lost due to exocytosis. Mutants where exocytosis is severely obstructed may take longer to reach a plateau^29^.

When treating cells with inhibitors that are effective against macropinocytosis in *Dictyostelium *(set up as in Figure 4A), the dextran internalized in 1 h will go down to almost nothing at higher inhibitor concentrations in the majority of cases (Figure 4B). Some inhibitors may not be 100% effective, however, *e.g.* nocodazole only inhibits up to 50% of fluid uptake by macropinocytosis when added acutely (Figure 4C). If the inhibitors are not effective, the cells will internalize a similar amount of dextran as the control, and a decrease in fluorescence will not be seen. This technique allows a large range of different inhibitors and inhibitor concentrations to be screened for effects on fluid uptake by macropinocytosis very quickly, reducing the time spent optimizing the inhibitor treatment.

**Figure:**
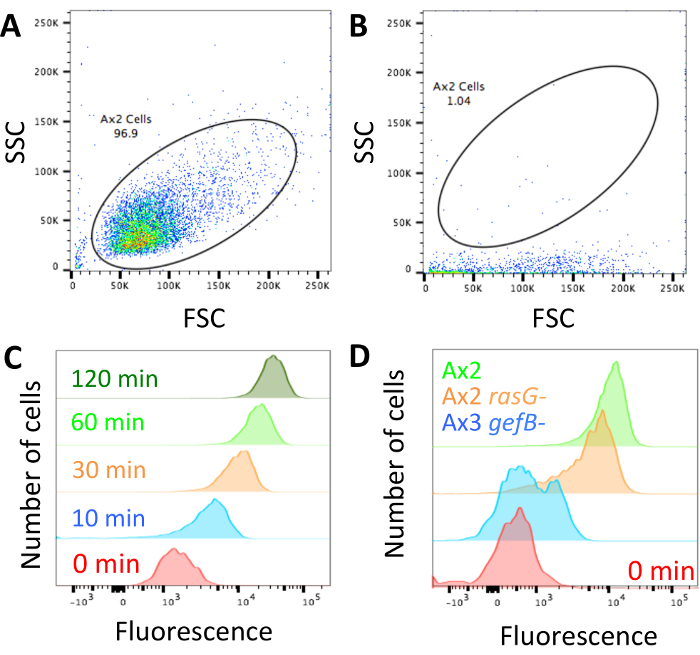

**Figure 2: Set up of flow cytometer and representative data. (A)** The forward scatter (FSC) and side scatter (SSC) profiles of cells should be set so the cells can be easily distinguished. An example of how Ax2 cells should look is shown. **(B)** If the flow cytometer is blocked, as in this example, the cells have very low side scatter. The laser will not excite the fluorophores properly and the machine should be unblocked before continuing. Any data obtained while the machine was blocked should be discarded. **(C) **The fluorescence should be set so that a 0 min uptake sample has low fluorescence, which increases when cells have been incubated for longer in the fluorescent medium, as shown in this example taken from Williams & Kay 2018^24^. **(D) **Examples of cells that have been incubated with TRITC dextran for 1 h with normal macropinocytosis (Ax2, green), reduced macropinocytosis (Ax2 *rasG-*, HM1726^14^, orange) and abolished macropinocytosis (Ax3 *gefB-*, HM1776^28^, blue). Please click here to view a larger version of this figure.

**Figure:**
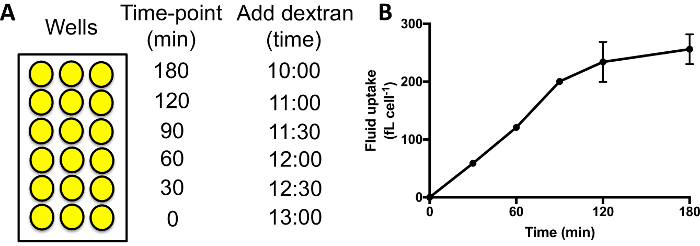

**Figure 3: Performing fluid uptake time-courses in 96-well plates. (A)** Dextran should be added to each set of samples sequentially, with the same finish time. Then wash the wells, detach and measure the internalized fluorescence by flow cytometry. Example times to add the dextran are shown here. **(B) **Fluid uptake time-course of Ax2 cells performed in 96-well plates. Taken from Williams & Kay 2018^24^, error bars show the standard error of three independent experiments. Please click here to view a larger version of this figure.

**Figure:**
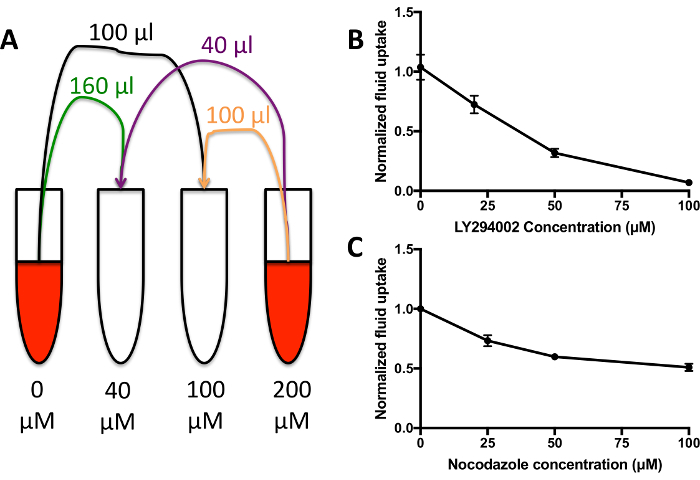

**Figure 4: Fluid uptake dose response curves. (A)** Add the compound of interest, in this case the PI3K inhibitor LY294002, to HL5 containing 1 mg/mL TRITC-dextran at **double** the desired final maximum concentration. Mix with HL5 growth medium + 1 mg/mL dextran containing vehicle alone in various proportions to generate a dilution series of 200 µL medium per condition. Add to wells as normal for 1 h before washing and measuring internalized fluorescence. **(B) **Fluid uptake dose response curve for Ax2 cells incubated with the LY294002-containing medium from **A**. Adapted from Williams & Kay 2018^24^. Fluid uptake is normalized to an untreated control. Error bars show the standard error of three independent experiments. **(C) **Fluid uptake dose response curve for Ax2 cells incubated with the nocodazole. Adapted from Williams & Kay 2018^24^. Fluid uptake is normalized to an untreated control. Error bars show the standard error of three independent experiments. Please click here to view a larger version of this figure.

## Discussion

Whereas other methods to assess fluid uptake are low throughput, washing the cells *in situ *and the use of sodium azide to detach cells are the critical steps in this method, which allow high-throughput measurement of macropinocytosis, membrane uptake, or phagocytosis by *Dictyostelium*. As the cells are attached to a surface and the medium is not, they can be left attached while the medium around them is first thrown off and then changed by immersion in buffer and thrown off again. Sodium azide, which depletes cellular ATP and depolarizes the membrane^30^, is then used to detach the cells, and also prevents exocytosis without affecting cell viability^24^.

While using flow cytometry to measure macropinocytosis by *Dictyostelium* gives a very accurate measurement of fluid uptake very quickly, to establish the reason why a particular strain or condition has altered fluid uptake, further investigation using microscopy is required^24^. It should also be noted that previously published results have, in some cases, shown a difference in fluid uptake by mutant strains grown either on a surface (as in this case), or in shaking suspension (as in the standard protocol)^31^. Using this method may mean that, in rare cases, apparent fluid uptake defects are missed. Additionally, when measuring phagocytosis, only low concentrations of particles can be used. The maximum rate of phagocytosis that can be determined with this technique is far below the real maximum, although it is still possible to measure relevant differences in phagocytosis between strains and conditions^24^. To determine the maximum rate of phagocytosis, uptake must be measured in shaking suspension by an alternative protocol^27^. Cells that have phagocytosed beads have increased side scatter, so this should be corrected for accordingly when setting up the flow cytometer.

Flow cytometry can be used to measure fluid uptake in mammalian cells^32^, however the higher proportion of fluid phase uptake by other endocytic pathways than seen in *Dictyostelium* is a concern. In addition, cells are typically detached using trypsin at 37 °C, allowing further endocytic progression of internalized dextran. Ice-cold sodium azide does not cause macrophages to detach from a surface (Williams, unpublished observation), making this technique not applicable to mammalian cells without further optimization.

High throughput measurement of macropinocytosis has the potential to be used to screen quickly and cheaply for the effects of inhibitors, genetic mutation or gene knockdown on *Dictyostelium *cells. Mutants should always be compared to their direct parent only. If the reader has no prior preference for *Dictyostelium* strain, non-axenic strains such as DdB or NC4 are more "wild-type" than axenic ones and can be manipulated as effectively as axenic strains^33^. Otherwise, Ax2 strains are the axenic strains with the fewest genome duplications^34^, while many strains of Ax4 are Talin A knockouts and should be avoided if possible^23^. Most previously published strains can be ordered from the Dicty Stock Center^35^.

This technique allows greater investigative possibilities than was previously possible into the effects of different conditions, inhibitors and mutations on macropinocytosis by *Dictyostelium*.

## Disclosures

The authors have no conflicts of interest to disclose.
